# The Relationship Between the Use of Mobile Social Media and Subjective Well-Being: The Mediating Effect of Boredom Proneness

**DOI:** 10.3389/fpsyg.2020.568492

**Published:** 2021-01-13

**Authors:** Jie Bai, Kunyu Mo, Yue Peng, Wenxuan Hao, Yuanshan Qu, Xiuya Lei, Yang Yang

**Affiliations:** Department of Psychology, School of Humanities and Social Science, Beijing Forestry University, Beijing, China

**Keywords:** short video, problematic use of mobile social-media, subjective well-being/SWB, the boredom proneness, network environment

## Abstract

**Objective:**

This study took users of short-form mobile videos as research participants to explore the role of their boredom proneness in mediating the relationship between the use of mobile social media (UMSM) and subjective well-being (SWB).

**Methods:**

A sample of 656 users was evaluated by the Problematic Mobile Social Media Usage Assessment Questionnaire, General Well-Being Schedule, and Boredom Proneness Scale.

**Results:**

Firstly, significant interactions were found between monthly living expenses and the UMSM of the participants, which were recognized as factors affecting SWB. Secondly, the level of living expenses had little effect on the high-level and low-level groups of the UMSM but imposed a significant impact on the medium-level group. Thirdly, the UMSM showed an influence that could positively predict boredom; both the UMSM and boredom demonstrated a negative predictive effect on SWB.

**Conclusion:**

The findings indicate that the inappropriate use of mobile social media negatively affects users’ subjective well-being; boredom partially mediated the relationship between the use of mobile social media and SWB.

## Introduction

In recent years, mobile social media have been used by more and more people due to its convenience. Although online social activities have become a supplement to the offline social life to a certain extent, excessive dependence on the Internet would inevitably induce more or less negative effects on the users (Jiang, 2018a). For instance, a decrease in subjective well-being was reported in individuals with Internet addiction (AFROZ, 2016; Mei et al., 2016; Nie et al., 2016; Koç, 2017; Suresh et al., 2018).

### Subjective Well-Being and the Use of Mobile Social Media

Subjective well-being (SWB) is a subjective, holistic, and relative indicator, which is widely used in psychological research as an overall assessment of the quality of life (Diener, 1984). SWB is also regarded as one of the standards for measuring mental health. People with high-level SWB could experience a higher level of self-esteem (Joshanloo and Daemi, 2015) and be more tolerant of others (Datu, 2013). Several factors have been reported to be effective to influence SWB (Yamaguchi et al., 2013), such as individual personality traits, style of attribution, physical health, socioeconomic status, social support, etc. (Diener et al., 2013; López Ulloa et al., 2013; Zhang et al., 2014). Among the affecting factors, the critical role of social support has been repeatedly verified (Khan and Husain, 2010; Wang, 2014; Tian et al., 2015). When people obtained more social support, they experienced less loneliness and more happiness (Q. Tian, 2014). In contrast, people with less social support are likely to turn to mobile social media to get noticed (e.g., by posting a tweet and attracting comments). Some studies have shown that using mobile social media can strengthen the connection with others and provide social support, to help people enhance their SWB (Boyd and Ellison, 2007; Koroleva et al., 2011; Wenninger et al., 2018). However, with the advancement of research, it has been demonstrated that individuals who use mobile social media frequently are more inclined to develop addictive behaviors, which may cause a series of negative effects such as anxiety, depression, etc. (Labrague, 2014). The improper use of mobile social media imposes negative impacts on people both physically and psychologically, thereby affecting their SWB (Hanprathet et al., 2015; Hawi and Samaha, 2016). A negative correlation has been found between the levels of SWB and problematic use of the Internet (AFROZ, 2016; Mei et al., 2016; Nie et al., 2016; Koç, 2017; Suresh et al., 2018).

### Boredom Proneness and SWB

Boredom is a state of being weary and restless through lack of interest. Also, boredom proneness refers to a persistent personality trait reflecting how easy an individual is apt to feel bored (Farmer and Sundberg, 1986; Eastwood et al., 2012). The individual feels bored when the environment cannot provide enough emotional stimuli. One with higher boredom proneness is more possible to generate negative emotions, which may lead to depression, anxiety, loneliness, and lower levels of SWB (German and Latkin, 2012). Thus, individuals tend to look for something new and exciting from the environment to alleviate boredom (Skues et al., 2015). Once an individual becomes overdependent on a stimulus that was novel, the adaptation to the novelty can reduce the interest and lead to new boredom. On the contrary, boredom proneness also acts as a predictor of the overdependence on a certain object. It was suggested that individuals whose personality trait is easy to feel bored are more possible to indicate Internet addiction (Chaney and Blalock, 2006). Titilope (2014) examined the use of mobile phones from a psychosocial dimension and found that boredom proneness could significantly predict the degree of dependence on mobile phones in adolescents. Leung (2008) found that people who were more likely to be bored used mobile phones more frequently. So, the interplay between boredom proneness and excessive reliance on certain specific activities or tools appears mutual and complex. In the latest decade, the use of mobile social media keeps rising, owing to the increasing interest of people in the new product that integrates features of the Internet and mobile phone. Nevertheless, to answer the question whether the gradually unfolding dependence on mobile social media is associated with boredom proneness, further investigations are still necessary.

In addition, a negative correlation between boredom proneness and SWB has been revealed. Individuals manifesting a high degree of boredom tended to show negative emotions and a lower level of SWB (German and Latkin, 2012). The study by Wang et al. (2014) confirmed that boredom proneness could negatively predict SWB. Combined with the aforementioned evidence indicating the links between the use of mobile social media and SWB, the two variables and boredom proneness constitute a new psychological framework. Rosen et al. (2013) suggested that bored people utilized social networks to relieve their boredom. In the Internet environment, it seems feasible to reduce the boredom by using the Internet appropriately and consequently improve SWB (Rosen et al., 2013; Greyling, 2018). A number of investigations have also been carried out focusing on the relationship between Internet-based utility and negative emotions (Bozoglan et al., 2013; Banjanin et al., 2015; Chen et al., 2019), whereas few studies have explored the role of boredom in the interrelationship between Internet use and SWB.

### Aims and Hypotheses

Some evidence has proven that social media imposed a significant effect on SWB (Uysal et al., 2013; Brooks, 2015). However, the mechanism underlying the interactions remains ambiguous (Nie et al., 2016). In our study, we surveyed users of short video who had habitual use of Internet-based social media, to figure out the pathways on how the individuals’ well-being is influenced by the improper use of social media and boredom proneness in the cyber world.

Given that boredom proneness is related to the use of social media and enables the prediction of the level of SWB, the current study raised the following hypotheses: (1) correlations exist among the use of mobile social media, boredom proneness, and SWB; (2) boredom proneness is a mediator variable between the problematic use of mobile social media and SWB, which means that the problematic use of mobile social media affects SWB *via* boredom proneness.

## Materials and Methods

### Ethics

The recruitment of participants for this study was approved by the Ethics Committee of the Department of Psychology, Beijing Forestry University. A survey was carried out with all the participants online or offline. All data were collected with the consent of the participants. Before the survey, the participants were informed about the research content and their rights.

### Participants

In this study, an online questionnaire survey platform called “Questionnaire Star”^1^, as well as offline questionnaires, was used to collect data on the use of mobile social media from short-video users. Due to the COVID-19 pandemic and the restriction of close social contact, the offline data collection could not continue. As a result, a mix of online and offline data collection was adopted.

After eliminating unqualified questionnaires (e.g., questionnaires that were filled out randomly), a total of 656 valid samples were collected, including 237 male samples (36.1%) and 419 female samples (63.9%).

### Measures

#### Problematic Use of Mobile Social Media

The Problematic Mobile Social Media Usage Assessment Questionnaire developed by Jiang (2018b) was used in this study. This questionnaire includes 20 questions, divided into five subdimensions, which are used to measure five different aspects of the use of mobile social media (UMSM). (1) *Viscosity increase* is used to measure the time length, frequency, and intensity of the use of mobile social media. For example, “Always extend the time of using mobile social-media without awareness” and “I have a certain dependence on mobile social-media, and sometimes cannot control the using time.” A higher score on this factor means that individuals use mobile social media for a longer time and more frequently and individuals are more dependent on mobile social media. (2) *Physiological damage* refers to the negative physical responses of individuals after excessive use of mobile social media, such as impaired vision, lack of sleep, shoulder pain, etc. A higher score on this factor means that the use of mobile social media has caused more serious physiological damage to individuals. To some certain extent, the improper use of mobile social media can be reflected by a physical condition. (3) *Omission anxiety* refers to the anxiety caused by individuals’ concerns about missing messages due to their inability to check their mobile social media in time. A higher score on this factor indicates a higher level of anxiety caused by an individual’s uncontrollable worry about missing information. This emotion could affect people’s concentration on their ongoing tasks. (4) *Cognitive failure* refers to the negative consequences of using mobile social media for cognition, such as memory loss and thinking stagnant. “Due to the convenience of mobile phones and mobile networks, I rarely remember things by myself, which made my memory gradually decline.” “Because of excessive dependence on the mobile social media, a great amount of information is no longer needed to be thought and processed by individuals, which dulls our mind and causes memory loss.” This is also a manifestation of excessive reliance on mobile social media. (5) *Guilt* is the feeling of being unable to complete an individual’s work or study schedule on time due to using mobile social media for a long time, for example, “I often regret wasting too much time on mobile social media.” This feeling may make the individual fall into constant self-blame and compunction. A higher score on this factor indicates that an individual feels guiltier for not completing a task on schedule due to unreasonable arrangement of using mobile social media. All items are scored from “1 = not at all” to “5 = completely true.” The higher total score of the UMSM represents the higher tendency in the problematic use of mobile social media. In this study, the internal consistency coefficient of the scale was 0.93, showing that this scale was highly reliable in the survey.

#### Subjective Well-Being

The Overall Happiness Scale revised by Duan (1996) was used in this study. The scale has 18 items, covering satisfaction and interest of life, energy, concerns about health, depressed or positive emotions, and control of emotions and behavior, as well as tension and relaxation. A higher score on this scale means a higher level of SWB. In this study, the internal consistency coefficient of the scale was 0.84, revealing its high reliability.

#### Boredom Proneness

The Boredom Proneness Scale was developed by Huang et al. (2010). The questionnaire has a total of 30 items, including two dimensions—external stimuli and internal stimuli. The external stimuli include four factors, and they are monotony, loneliness, tension, and restraint. On the other hand, the internal stimuli include two factors, and they are self-control and creativity. All items are scored from “1 = not at all” to “5 = completely true.” In this study, we only used the total score to measure the individual’s boredom proneness. Individuals showing a higher total score of boredom proneness are characterized by higher boredom proneness and the tendency to be bored easily. The internal consistency coefficient of the scale was 0.92 in the current study.

### Data Processing

All data were processed and analyzed by using statistical software SPSS 24.0. A series of analyses were implemented to check the systematic errors and explore the relationship among various psychological variables.

A common method bias test was conducted to find out whether the properties of the data in this study affected the results. The common method bias is a systematic error which can be attributed to several environmental factors, such as the experimental settings, the way how the participants answer the questionnaires, and so forth. These factors can enlarge the errors and bias the final results of the study. Therefore, the aim of the implementation of the common method bias effect testing was to confirm whether such systematic error exists in our collected dataset. According to the test method introduced by Zhou and Long (2004), the method of “separating the first common factor” was used to compare the model fitting degree before and after controlling the deviation of the common method.

After we eliminated the possibility of common method biases, we performed the variance tests to explore the significance in the use of mobile social media, SWB, and boredom proneness, influenced by differences in gender, age, daily short-video viewing duration, and daily mobile social media usage time.

The Pearson correlation analysis was conducted to examine the correlations between pairs of variables, followed by a stepwise regression analysis to explore the linear relationship between the five factors of the use of mobile social media and SWB.

We also used a two-factor ANOVA to analyze whether the main effects of demographic variables and the use of mobile social media exist to influence SWB, as well as the potential interaction between the two factors. Notably, among the demographic variables of the participants, we focused on the “monthly living expenses,” as we considered it an indicator of an individual’s economic status, which might be related to SWB. Finally, the PROCESS 2.1 program and bootstrap method were employed to verify the mediating effect of boredom proneness.

## Results

### Common Method Bias Test

The results of the single-factor test showed that for the 12 factors with eigenvalues greater than 1, the first factor explained 26.04% of the variation, which was lower than the critical value of 40%. Therefore, the common method deviation had little effect on the following analyses (see Supplementary Table 1).

### Descriptive Statistics and Difference Tests

Gender, age, daily viewing duration of short videos, and daily usage time of mobile social media were utilized as grouping variables for the short-video users in the *t*-tests or one-way ANOVA analyses, which were carried out to examine the differences caused by the demographic factors in UMSM, SWB, and boredom proneness. The results are shown in Tables 1–3 and Supplementary Tables 2–4.

**TABLE 1:** The results of difference analysis on UMSM.

Variable	Number	UMSM	*t*/*F*	*p*
**Gender**				
Male	237	3.28 ± 0.82	2.825**	0.005
Female	419	3.10 ± 0.78		
**Age**				
Under the age of 18	95	2.84 ± 0.78	11.382***	0.000
Aged 18–24	426	3.25 ± 0.76		
Aged 24 and above	135	3.10 ± 0.80		

UMSM, use of mobile social media; **correlation is significant at the 0.01 level (two tailed); ***correlation is significant at the 0.001 level (two tailed).

**TABLE 2:** The results of difference analysis on SWB.

Variable	Number	SWB	*t*/*F*	*p*
**Gender**				
Male	237	4.25 ± 0.70	−1.986*	0.047
Female	419	4.37 ± 0.77		
**Age**				
Under the age of 18	95	4.38 ± 0.78	2.418	0.09
Aged 18–24	426	4.28 ± 0.75		
Aged 24 and above	135	4.44 ± 0.74		

SWB, subjective well-being. *Correlation is significant at the 0.05 level (two tailed).

**TABLE 3:** The results of difference analysis on BP.

Variable	Number	BP	*t*/*F*	*p*
**Gender**				
Male	237	3.62 ± 0.92	4.377***	0.000
Female	419	3.29 ± 0.90		
**Age**				
Under the age of 18	95	3.66 ± 0.92	8.366***	0.000
Aged 18–24	426	3.43 ± 0.88		
Aged 24 and above	135	3.17 ± 0.99		

BP, boredom proneness. ***Correlation is significant at the 0.001 level (two tailed).

Males’ total scores of the UMSM and boredom proneness were higher than those of females (*t* = 2.825, *p* < 0.005; *t* = 4.377, *p* < 0.000); males’ SWB score was significantly lower than that of females (*t* = −1.986, *p* < 0.047).

Significant differences among different age groups were found in scores of both UMSM and boredom proneness (*F* = 11.382, *p* < 0.000; *F* = 8.366, *p* < 0.000). The following multiple comparisons showed that, regarding the UMSM scores, the group “Under the age of 18” had lower scores than the group “Aged 18–24” and the group “Aged 24 and above.”

For the scores of boredom proneness, the group “Under the age of 18” had higher scores than the group “Aged 18–24,” and the group “Aged 18–24” had higher scores than the group “Aged 24 and above.” That is, boredom proneness decreased with age.

### Correlation Among Boredom Proneness, UMSM, and SWB

This study used the Pearson correlation to analyze the relationship among boredom proneness, UMSM, and SWB. In Table 4, it suggested that SWB showed a significantly negative correlation with the UMSM; boredom proneness showed a significantly positive correlation with the UMSM and a significantly negative correlation with SWB.

**TABLE 4:** Correlation between variables.

	*M*	*SD*	1	2	3	4	5	6	7	8
(1) SWB	4.33	0.75	1							
(2) UMSM	3.16	0.80	−0.392***	1						
(3) Viscosity increase	3.49	0.93	−0.260***	0.801***	1					
(4) Physiological damage	2.99	0.95	−0.346***	0.847***	0.547***	1				
(5) Omission anxiety	3.06	0.99	−0.354***	0.851***	0.636***	0.635***	1			
(6) Cognitive failure	3.07	0.95	−0.343***	0.837***	0.527***	0.647***	0.652***	1		
(7) Guilt	3.17	1.19	−0.295***	0.698***	0.426***	0.528***	0.492***	0.610***	1	
(8) BP	3.41	0.92	−0.604***	0.420***	0.307***	0.341***	0.368***	0.383***	0.313***	1

UMSM, use of mobile social media; SWB, subjective well-being; BP, boredom proneness; ***correlation is significant at the 0.001 level (two tailed).

According to the total score of the UMSM, participants were divided into the high-level group (the top 27%), medium-level group (the middle 46%), and low-level group (the bottom 27%). The independent sample test was used to compare SWB and boredom proneness between groups of high and low levels. The results showed that the SWB of the low-level group was significantly higher than that of the high-level group (*t* = 9.996, *p* < 0.001), and the low-level group had significantly lower boredom proneness than the high-level group (*t* = −10.807, *p* < 0.001).

In the group with low-level UMSM, the Pearson correlation coefficient (PCC) between the UMSM and SWB was −0.200 (*p* < 0.01), and the PCC between the SWB and boredom proneness was −0.446 (*p* < 0.001). In the high-level group, the PCC between the UMSM and SWB was −0.162 (*p* < 0.05), the PCC between the UMSM and boredom proneness was 0.244 (*p* < 0.01), and the PCC between SWB and boredom proneness was −0.575 (*p* < 0.001). In the medium-score group, there was no correlation between the UMSM and SWB, and the PCC between the SWB and boredom proneness was −0.576 (*p* < 0.001) (Supplementary Tables 5–7).

### Main Effects and Interaction Analysis

A two-factor ANOVA analysis was implemented to explore the main effects of the UMSM and monthly living expenses on SWB, as well as the possible interaction between the two factors. Multiple levels of each factor were taken into the analysis. As a result, the main effect of the UMSM on SWB was significant (*F* = 43.77, *p* < 0.001); the main effect of monthly living expenses on SWB was not significant (*F* = 0.02, *p* > 0.05), whereas the interaction between the UMSM and monthly living expenses was significant (*F* = 3.943, *p* < 0.05).

**FIGURE 1:**
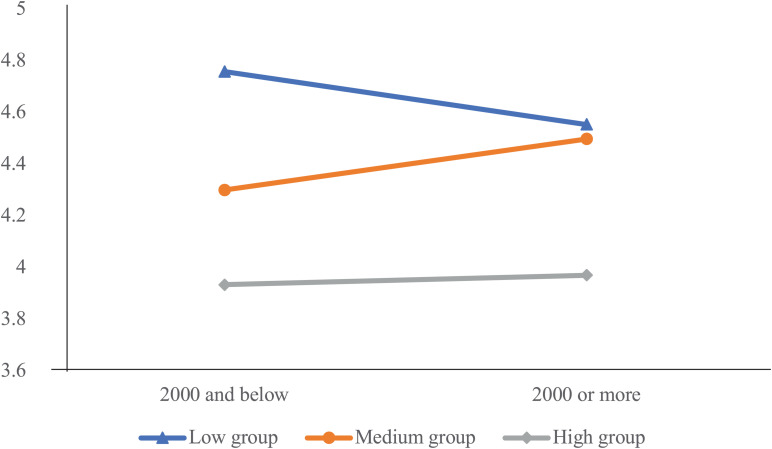
Interaction between living expense and mobile social media use on SWB. A 3 (use of mobile social media) * 2 (living expense) analysis of variance to show the interaction between the two factors.

Further simple effect analysis revealed that for the groups with high- and low-level UMSM, living expenses had no significant effect on SWB; for the group with medium-level UMSM, the SWB of participants reporting high living expenses (more than CNY 2,000) was higher than that of participants reporting low living expenses (less than CNY 2,000) (*p* < 0.05). The results demonstrated that different levels of living expenses are related to discrepancies in SWB, which is valid only for the group showing medium-level UMSM (see Figure 1).

### Regression Analysis of the UMSM and SWB

Multiple stepwise regression analysis was performed based on the correlation analysis. The SWB was used as the dependent variable, and the five factors constituting the UMSM were used as the predictor variables. As shown in Table 5, three of the five factors—omission anxiety, physiological damage, and guilt—exerted negative predictive effects on the SWB of short-video users (β = −0.148, *p* < 0.001; β = −0.128, *p* < 0.01; β = −0.072, *p* < 0.01); the contribution rates reached 12.4, 14.8, and 15.5%, respectively.

**TABLE 5:** Regression analysis of the use of mobile social media and SWB.

Dependent variable	Independent variable	*R*	*R*^2^	Δ*R*^2^	*F*	β	Beta	*t*
SWB	Omission anxiety	0.354	0.124	0.126	93.877	−0.148	−0.195	−4.085***
	Physiological damage	0.388	0.148	0.025	57.717	−0.128	−0.162	−3.309**
	Guilt	0.399	0.155	0.009	41.081	−0.072	−0.113	−2.605**

SWB, subjective well-being; **correlation is significant at the 0.01 level (two tailed); ***correlation is significant at the 0.001 level (two tailed).

### The Mediating Effect of Boredom Proneness

**FIGURE 2:**
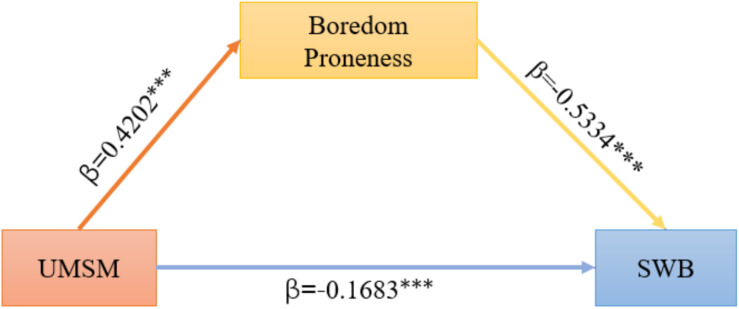
The mediating effect of boredom proneness. Test of the mediating role of boredom proneness between the use of mobile social media and subjective well-being. ^∗^*p* < 0.05, ^∗∗^*p* < 0.01, ^∗∗∗^*p* < 0.001. UMSM, use of mobile social media; SWB, subjective well-being; ^∗^correlation is significant at the 0.05 level (two tailed); ^∗∗^correlation is significant at the 0.01 level (two tailed); ^∗∗∗^correlation is significant at the 0.001 level (two tailed).

On basis of the test method proposed by Ye and Wen (2013), we examined the mediating effect of boredom proneness on the relationship between the UMSM and SWB. The result showed that the UMSM enabled a significantly negative prediction of SWB (β = −0.1683, *p* < 0.001). Boredom proneness allowed a significantly negative prediction of SWB as well (β = −0.5334, *p* < 0.001). The UMSM gave rise to a significantly positive prediction of boredom proneness (β = 0.4202, *p* < 0.001), indicating that boredom proneness played a partial mediating role in the interplay between the UMSM and SWB. In summary, the mediation model hypothesized in this study was supported (see Figure 2).

In order to verify the model, this study used the PROCESS program compiled by Hayes to conduct the bootstrap test (5,000 times). The result showed that the confidence interval of 95% for the UMSM to influence SWB through boredom proneness was [−0.2848, −0.1728] (see Table 6).

**TABLE 6:** Test of the mediating effect of the use of mobile social media, boredom proneness, and SWB.

Mediator	Effect	Effect size	Effect ratio	Boot SE	BootCI LL	BootCI UL
Boredom proneness	Total effect	−0.3924***		0.0360	−0.4630	−0.3218
	Direct effect	−0.1683***		0.0337	−0.2345	−0.1021
	Indirect effect	−0.2241***	57.11%	0.0284	−0.2848	−0.1728

***Correlation is significant at the 0.001 level (two tailed).

## Discussion

### Demographic Analysis of the UMSM, SWB, and Boredom Tendency

Results of the descriptive statistics showed that there were gender differences in the UMSM. Males’ scores on the UMSM were significantly higher than females’ scores. That is to say, males tend to have more problematic use of mobile social media than females. This was consistent with the result of previous research (Tomaszek and Muchacka-Cymerman, 2019). On the overall score of SWB, females’ score was significantly higher than that of males, which may be caused by the different motivations of males and females on using mobile social media and their different preferences on specific functions when using it. Males are more inclined to use mobile social media for work-related instrumental purposes, while females use it more as a way to communicate with important people, maintain contact, or perform some entertainment activities to achieve satisfaction (Walsh et al., 2011). This is the reason why females’ SWB is higher. At the same time, this study found that the longer time individuals use mobile social media, the lower SWB they could perceive.

Moreover, there were differences in the scores of the UMSM and boredom proneness of different age groups. There was no difference in SWB among different age groups, which showed that SWB of short-video users was not affected by age. That is, in the long run, SWB is a relatively stable measure (Diener, 1984). For the scores of the UMSM, the users in the “Under the age of 18” group showed lower scores than those in the “Aged 18–24” group, and the users in the “Aged 18–24” group presented lower scores than those in the “Aged 24 and above” group. Overall, the tendency of problematic use of mobile social media increases with age. As to the scores of boredom proneness, the “Under the age of 18” group had higher scores than the “Aged 18–24” group, and the “Aged 18–24” group had higher scores than the “Aged 24 and above” group. That is, boredom proneness decreases with age. In China, most of the teenagers under the age of 18 are trapped in academic stress and do not have enough time for extracurricular activities out of school, particularly using mobile phones, which is usually limited under the supervision of parents. When staying in a highly constrained environment, people often feel more bored (Chin et al., 2017). Besides, numerous students feel caught by rigid routines from which they cannot escape (Daschmann et al., 2011). As the students grow up, the time at their disposal increases proportionally, by which they are able to engage in more activities according to their own ideas, to enrich their lives.

The result of the main effect and simple effect analyses showed that after dividing the tendency of problematic use of mobile social media into three groups of high, medium, and low level, it can be found that there were significant differences in the impact of living expenses on SWB among the different groups. In this study, most of the short-video users were from 18 to 24 years old, accounting for 64.93% of the total sample. This group of participants was primarily comprised of college students, who had limited income, so the CNY 2,000 was determined as the standard for dividing samples into high- and low-level economic status. An interaction effect influencing SWB was found in our study between two factors—the UMSM and the economic status of the participants. In general, a co-variation was exhibited between economic status and SWB in the medium-level UMSM group, where the SWB level of people with living expenses above CNY 2,000 was higher than that of people with living expenses below CNY 2,000. It is consistent with the findings of the positive correlation between income and SWB (Clark et al., 2005; Carroll et al., 2007). However, an inconformity was revealed in the low- and high-level UMSM groups, in which the SWB of participants was independent of their economic status. Within the low-level UMSM group that showed the greatest SWB, no significant difference was identified between the high-expense and low-expense subgroups in this degree of SWB. This might be caused by the “marginal utility” which implies that the benefit to one of an additional unit of happiness is inversely related to the number of units of happiness he already owns. The satisfaction derived from higher income barely progresses further in the people who are already satisfied with their economic status (Kahneman, 2006). Therefore, the change in SWB was not significant when economic status was taken as the independent variable for the high-SWB (low-UMSM) group. Within the high-level UMSM group demonstrating the lowest SWB, no apparent difference was revealed between the subgroups with discrepant economic status. This result is inconsistent with previous evidence showing that lower-level economic status exacerbated the distress of people with poorer SWB caused by life events, such as divorce, illness, and being alone (Kahneman and Deaton, 2010). In the present study, differences in economic status did not affect the degree of SWB in the individuals showing less happiness that was associated with their problematic use of mobile social media. The result implies that low-level SWB linked to the UMSM differs from that induced by stress events or life pressure, which reflects the complexity among the UMSM, economic status, and SWB. To unveil the panorama, more in-depth investigations are needed.

### Impact of UMSM on SWB

This study confirmed that mobile social media was an important variable for predicting SWB. The higher the tendency of the problematic use of mobile social media, the lower the SWB which was perceived by individuals. This result showed that the use of mobile social media imposed a significant effect on SWB and enabled a significantly negative prediction of SWB. This was consistent with previous research results (Uysal et al., 2013; Brooks, 2015). On the one hand, the social comparison theory points out that the happiness of an individual results from comparing himself with other individuals. It will reduce an individual’s subjective happiness when he/she compares himself/herself with a happier person (Festinger, 1954). The convenience of mobile social media makes it easier for individuals to access information about what is happening in their social community by browsing social media platforms. People are more inclined to post interesting and delightful life stories *via* mobile social media to build up popular personal images. When such stories are captured by the audience, the unconscious comparison of themselves with the “leading characters” in stories may make them feel gloomy, which consequently reduces their level of SWB. More than 50% of social media users think that their friends are happier than themselves (Tamir and Mitchell, 2012). On the other hand, when individuals need to handle negative life events, they are more likely to adopt a coping style of avoidance (Wu et al., 2014) and gain more positive emotions through mobile social media. However, the prolonged use of mobile social media is more likely to cause individuals to feel guilty and fall into self-blame. This negative emotional experience can also reduce an individual’s sense of well-being (Katana et al., 2019).

Furthermore, three of the five subdimensions constituting the UMSM—physiological damage, omission anxiety, and guilt—had negative predictive effects on SWB, which was revealed by the stepwise regression analysis. This might be due to the fact that physiological damage caused individuals to develop excessive worries about their health. Both emotional distress and physical discomfort can affect quality of life (Estévez-López et al., 2015). Therefore, the worries resulted in a decline of SWB. Omission anxiety refers to unrest when one is unable to view the information in time and worry about missing messages. This emotion could reduce the individual’s level of SWB (Malone and Wachholtz, 2017). When individuals are immersed in mobile social media, they might unconsciously increase the time and frequency of the use of mobile social media, and this results in failure to complete relevant work or achieve study goals. This is a situation that can lead to self-blame and self-criticism, which can also decrease well-being (Przybylski et al., 2013). These results suggest that when individuals have more awareness of their discomfort related to the use of mobile social media, whatever it is from the physical aspect (e.g., physical damage) or the psychological aspect (e.g., guilt), the level of SWB tends to decrease. On the contrary, if the individuals are not aware of these, their SWB level will not be affected.

### The Mediating Effect of Boredom Proneness

This study showed that boredom proneness played a partial mediating role in the interaction between the UMSM and SWB. Our findings provide evidence to explain the mechanism underlying the interplay between the inappropriate use of the Internet and SWB reported in previous researches (Boyd and Ellison, 2007; Koroleva et al., 2011; Wenninger et al., 2018). The fundamental reason why boredom proneness is involved in mediation is that boredom *per se* influences SWB. Another explanation is the weakened attention induced by the improper use of social media.

The excessive use of the Internet has been linked to attention deficits (Kawabe et al., 2019). When a person is overly reliant on the use of mobile social media, his/her attentiveness is vulnerable. Meanwhile, boredom is sometimes led by the absence of attention to the current goals (Hunter and Eastwood, 2016). Individuals with high boredom proneness are more inclined to experience boredom. In a state of boredom, a lack of attention drives negative emotions, and when attention is not fully engaged, the activities would be negatively treated, which results in a negative emotional state and affects the level of SWB (Fahlman et al., 2011).

## Limitations

This study also has some limitations. Firstly, according to the collected questionnaire results, most of the short-video users were from 18 to 24 years old. In future research, the age range of the participants could be expanded, and more in-depth research could be conducted based on a population with different age groups. Secondly, according to the results, this study divided the monthly living expenses into above CNY 2,000 and below CNY 2,000. In future studies, the impact of living expenses on mobile social media could be further explored by considering people from different occupations and collecting more detailed data related to living expenses. Thirdly, the survey adopted the self-reported approach, which may be affected by the social desirability effect, resulting in biases in the measurement results. Fourthly, this study is a preliminary exploration, and the factor *usage style* of short-video users could be taken into consideration in the future study. Finally, this study showed that omission anxiety, physical damage, and guilt involved in the use of mobile social media imposed a negative predictive effect on SWB. In future research, more effort could be made to explore the mechanism underpinning the relations among the relevant factors.

## Conclusion

Previous investigations have revealed the negative impact of the inappropriate use of mobile social media on one’s SWB. However, the mechanism underlying the relationship between the two variables is unclear. The current study focused on another variable—boredom proneness, and figured out its mediating role between the use of mobile social media and SWB. This study not only provides new evidence to verify the influence path proposed in previous studies but also demonstrated the principle of how the dynamic model works.

## Data Availability Statement

The raw data supporting the conclusions of this article will be made available by the authors, without undue reservation.

## Ethics Statement

This study involved human participants, and has been reviewed and approved by Ethics Committee of Department of Psychology, Beijing Forestry University. The ethics committee waived the requirement for written informed consent. However, written informed consent was implied via completion of the questionnaire.

## Author Contributions

JB, XL, and YY designed the study. JB, KM, and YP collected the data. JB, WH, and YQ analyzed the data. JB and YY wrote the manuscript. JB, YY, XL, KM, YP, WH, and YQ revised the manuscript. All authors contributed to the article and approved the submitted version.

## Conflict of Interest

The authors declare that the research was conducted in the absence of any commercial or financial relationships that could be construed as a potential conflict of interest.

## Footnotes

1 https://www.wjx.cn/
