# Supplementary material for: The Relationship Between the Use of Mobile Social Media and Subjective Well-Being: The Mediating Effect of Boredom Proneness
Source: Front Psychol. 2021 Jan 13;11:568492. doi: 10.3389/fpsyg.2020.568492 (PMC7874194; doi:10.3389/fpsyg.2020.568492)
Supplement: Supplementary file 1 [file Data_Sheet_1.pdf]

Supplementary Materials

Figure S1.

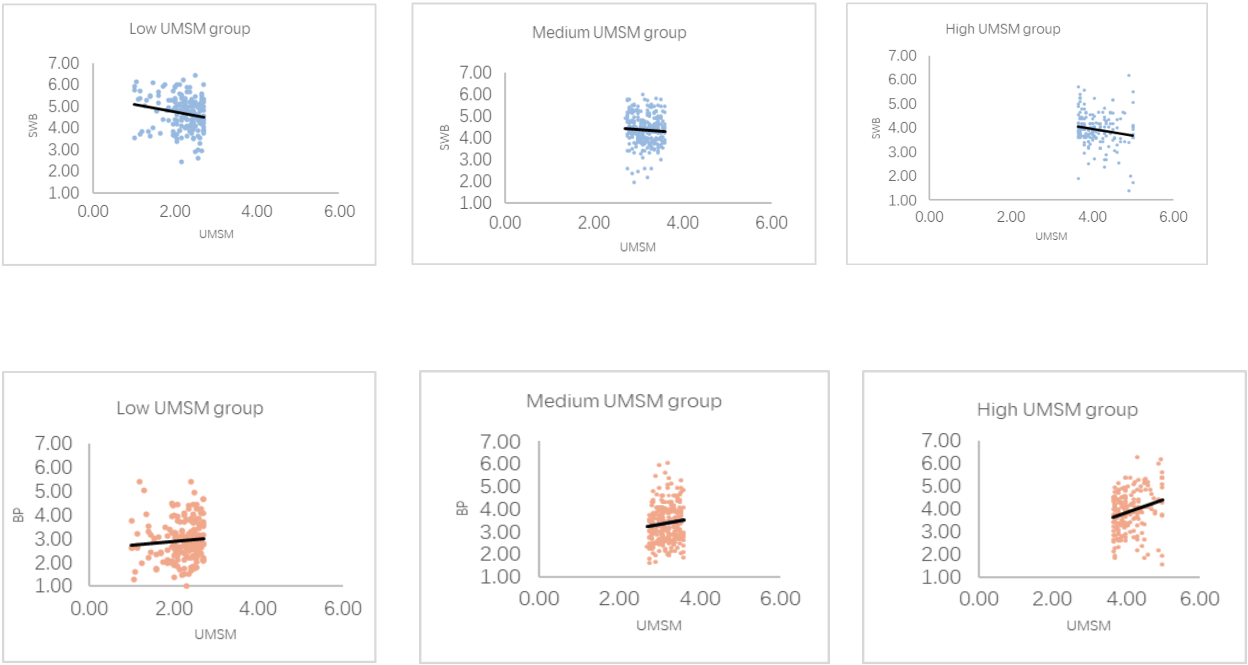

Figure S1. Scatter plot of the correlation of variable under UMSM grouping. UMSM= use of mobile social-media; SWB= subjective well-being; BP= boredom proneness

Table S1

Table S1. Total variance explained

Component	Initial Eigenvalues			Extraction Sums of Squared Loadings		
	Total	% of Variance	Cumulative %	Total	% of Variance	Cumulative
1	17.707	26.040	26.040	17.707	26.040	26.040
2	6.240	9.177	35.217	6.240	9.177	35.217
3	4.873	7.166	42.383	4.873	7.166	42.383
4	3.410	5.015	47.398	3.410	5.015	47.398
5	2.391	3.516	50.914	2.391	3.516	50.914
6	2.037	2.996	53.910	2.037	2.996	53.910
7	1.721	2.531	56.441	1.721	2.531	56.441
8	1.387	2.040	58.482	1.387	2.040	58.482
9	1.316	1.936	60.417	1.316	1.936	60.417
10	1.255	1.846	62.263	1.255	1.846	62.263
11	1.178	1.732	63.996	1.178	1.732	63.996
12	1.027	1.510	65.506	1.027	1.510	65.506

Table S2

Table S2. The results of difference analysis on UMSM

Variable	Number	UMSM	t/F	P
**Time of viewing short video (daily)**				
within 30 minutes	168	2.99±0.77	6.682***	0.000
30-60 minutes	163	3.08±0.78		
1-2 hours	168	3.26±0.79		
2 hours and above	157	3.34±0.82		
**Use time of mobile social media (daily)**				
2 hours and below	139	2.86±0.81	10.866***	0.000
2-3 hours	152	3.11±0.75		
3-5 hours	167	3.29±0.77		
5 hours and above	198	3.30±0.80		

Note. UMSM= use of mobile social-media; ***Correlation is significant at the .001 level (two tailed).

There is a significant difference in scores of the use of mobile social media on the time of viewing short video (daily (F=6.682, p<0.000). Further multiple comparisons afterwards found that in terms of the use of mobile social-media, the scores of those whose Time of viewing short video (daily) is less than 30 minutes will be significantly lower than that of people who watch1-2 hours and 2 hours and above, the scores of those who watch 30-60 minutes is significantly lower than that of people who watch1-2 hours and 2 hours and above.

There is a significant difference in scores of the use of mobile social media due to Use time of mobile social media (daily (F=10.866, p<0.000; F=2.73, p<0.043). Further multiple comparisons afterwards found that the subjective well-being of people who used social media for 2 hours or less is higher than that of people who used social media for 5 hours or more.

Table S3

Table S3. The results of difference analysis on SWB

Variable	Number	SWB	t/F	P
**Time of viewing short video (daily)**				
within 30 minutes	168	4.36±0.74	2.089	0.100
30-60 minutes	163	4.40±0.67		
1-2 hours	168	4.33±0.79		
2 hours and above	157	4.21±0.79		
**Use time of mobile social media (daily)**				
2 hours and below	139	4.46±0.75	2.731*	0.043
2-3 hours	152	4.36±0.69		
3-5 hours	167	4.31±0.76		
5 hours and above	198	4.23±0.78		

Note. SWB= subjective well-being. *Correlation is significant at the .05 level (two tailed).

There is a significant difference in scores of the use of subjective well-being due to different time lengths of the use time of mobile social media (daily) ($F=2.73$, $p<0.043$). Further multiple comparisons afterwards found that the subjective well-being of people who used social media for 2 hours or less is higher than that of people who used social media for 5 hours or more.

Table S4

Table S4. The results of difference analysis on BP

Variable	Number	BP	t/F	P
**Time of viewing short video (daily)**				
within 30 minutes	168	3.28±0.93	6.358***	0.000
30-60 minutes	163	3.28±0.84		
1-2 hours	168	3.42±0.91		
2 hours and above	157	3.67±0.96		
**Use time of mobile social media (daily)**				
2 hours and below	139	3.30±0.95	1.824	0.141
2-3 hours	152	3.34±0.82		
3-5 hours	167	3.44±0.93		
5 hours and above	198	3.51±0.96		

Note. BP= boredom proneness. ***Correlation is significant at the .001 level (two tailed).

There is a significant difference in scores of boredom proneness on the time of viewing short video (daily) (F=6.358, p<0.000). In terms of boredom proneness, the scores of people whose the time of viewing short video (daily) is less than 30 minutes, 30-60 minutes, and 1-2 hours are significantly lower than that of people who watch 2 hours and above. Overall, those users who watched short videos for more than 2 hours experience a higher degree of boredom proneness

Table S5

Table S5. Correlation matrix of various variables in low-level UMSM group

	UMSM	SWB	BP
UMSM	1		
SWB	-.200**	1	
BP	0.090	-.446***	1

Note. UMSM= use of mobile social-media; SWB= subjective well-being; BP= boredom proneness; **Correlation is significant at the .01 level (two tailed). ***Correlation is significant at the .001 level (two tailed).

In the low-level group of mobile social media usage, SWB and UMSM are significantly negatively correlated, and BP is significantly negatively correlated, while BP and UMSM are not significantly correlated.

Table S6

Table S6. Correlation matrix of various variables in medium-level UMSM group

	UMSM	SWB	BP
UMSM	1		
SWB	-0.052	1	
BP	0.098	-.576***	1

Note. UMSM= use of mobile social-media; SWB= subjective well-being; BP= boredom proneness; ***Correlation is significant at the .001 level (two tailed).

In the medium-level group of mobile social media use, SWB and UMSM are significantly negatively correlated with BP; while BP and UMSM are not significantly correlated.

Table S7

Table S7. Correlation matrix of various variables in high-level UMSM group

	UMSM	SWB	BP
UMSM	1		
SWB	-.162*	1	
BP	.244**	-.575***	1

Note. UMSM= use of mobile social-media; SWB= subjective well-being; BP= boredom proneness; *Correlation is significant at the .05 level (two tailed); **Correlation is significant at the .01 level (two tailed). ***Correlation is significant at the .001 level (two tailed).

In the high-level group of mobile social media use, SWB and UMSM are significantly negatively correlated, and BP is significantly negatively correlated; while BP and UMSM are significantly negatively correlated.

Table S8

Table S8. Re-analysis: the results of difference analysis on UMSM

Variable	Number	UMSM	t/F	P
**Gender**				
Male	237	3.28±0.82	10.072***	0.000
Female	237	2.63±0.56		
**Age**				
Under 18	84	2.76±0.78	4.155*	0.016
Age of 18-24	285	3.03±0.75		
Aged 24 and above	105	2.90.±0.80		
**Time of viewing short video (daily)**				
within 30 minutes	111	2.70±0.78	7.626***	0.000
30-60 minutes	107	2.89±0.73		
1-2 hours	123	3.13±0.77		
2 hours and above	133	3.05±0.75		
**Use time of mobile social media (daily)**				
2 hours and below	128	2.77±0.69	5.228**	0.001
2-3 hours	124	2.92±0.76		
3-5 hours	116	3.03±0.75		
5 hours and above	106	3.14±0.85		

Note. UMSM= use of mobile social-media; *Correlation is significant at the .05 level (two tailed); **Correlation is significant at the .01 level (two tailed). ***Correlation is significant at the .001 level (two tailed).

Table S9

Table S9. Re-analysis: the results of difference analysis on SWB

Variable	Number	SWB	t/F	P
**Gender**				
Male	237	4.25±0.70	-2.948**	0.003
Female	237	4.46±0.80		
**Age**				
Under 18	84	4.36±0.80	1.609	0.201
Age of 18-24	285	4.31±0.77		
Aged 24 and above	105	4.47±0.70		
**Time of viewing short video (daily)**				
within 30 minutes	111	4.47±0.78	2.639	0.049
30-60 minutes	107	4.41±0.69		
1-2 hours	123	4.35±0.72		
2 hours and above	133	4.21±0.82		
**Use time of mobile social media (daily)**				
2 hours and below	139	4.40±0.74	2.123	0.096
2-3 hours	152	4.44±0.65		
3-5 hours	167	4.35±0.79		
5 hours and above	198	4.20±0.85		

Note. SWB= subjective well-being. **Correlation is significant at the .01 level (two tailed).

Table S10

Table S10. Re-analysis: the results of difference analysis on BP

Variable	Number	BP	t/F	P
**Gender**				
Male	237	3.62±0.92	4.124***	0.000
Female	237	3.27±0.88		
**Age**				
Under 18	84	3.76±0.89	11.523***	0.000
Age of 18-24	285	3.46±0.89		
Aged 24 and above	105	3.14±0.95		
**Time of viewing short video (daily)**				
within 30 minutes	111	3.35±0.97	1.075	0.359
30-60 minutes	107	3.39±0.83		
1-2 hours	123	3.47±0.90		
2 hours and above	133	3.55±0.96		
**Use time of mobile social media (daily)**				
2 hours and below	139	3.31±0.94	5.004***	0.002
2-3 hours	152	3.33±0.82		
3-5 hours	167	3.46±0.87		
5 hours and above	198	3.73±1.00		

Note. BP= boredom proneness. ***Correlation is significant at the .001 level (two tailed).

Table S11

Table S11. Re-analysis: correlation between variables

	M	SD	1	2	3	4	5	6	7	8
1 SWB	2.95	0.77	1							
2 UMSM	4.35	0.76	-0.390***	1						
3 viscosity increase	3.28	0.93	-0.253***	0.786***	1					
4 physiological damage	2.77	0.92	-0.351***	0.834***	0.511***	1				
5 omission anxiety	2.86	0.96	-0.346***	0.851***	0.607***	0.627***	1			
6 cognitive failure	2.88	0.93	-0.329***	0.825***	0.504***	0.618***	0.650***	1		
7 guilt	2.95	1.43	-0.289***	0.681***	0.396***	0.501***	0.495***	0.563***	1	
8 BP	3.44	0.92	-0.604***	0.461***	0.333***	0.378***	0.388***	0.399***	0.357***	1

Note. UMSM= use of mobile social-media; SWB= subjective well-being; BP= boredom proneness; ***Correlation is significant at the 0.001 level (two tailed).

Table S12

Table S12. Re-analysis: correlation matrix of various variables in low-level UMSM group

	UMSM	SWB	BP
UMSM	1		
SWB	-.200**	1	
BP	0.090	-.446***	1

Note. UMSM= use of mobile social-media; SWB= subjective well-being; BP= boredom proneness; **Correlation is significant at the .01 level (two tailed). ***Correlation is significant at the .001 level (two tailed).

Table S13

Table S13. Re-analysis: correlation matrix of various variables in medium-level UMSM group

	UMSM	SWB	BP
UMSM	1		
SWB	-0.016	1	
BP	0.082	-.542***	1

Note. UMSM= use of mobile social-media; SWB= subjective well-being; BP= boredom proneness; ***Correlation is significant at the .001 level (two tailed).

Table S14

Table S14. Re-analysis: correlation matrix of various variables in high-level UMSM group

	UMSM	SWB	BP
UMSM	1		
SWB	-.217*	1	
BP	.238*	-.662***	1

Note. UMSM= use of mobile social-media; SWB= subjective well-being; BP= boredom proneness; *Correlation is significant at the .05 level (two tailed); ***Correlation is significant at the .001 level (two tailed).

Table S15

Table S15. Re-analysis: regression analysis of use of mobile social-media and SWB

Dependent variable	Independent variable	R	R^2	$\triangle R^2$	F	β	Beta	t
SWB	Physiological damage	0.351	0.123	0.121	66.278	-0.154	-0.187	-3.296**
	Omisson anxiety	0.386	0.149	0.146	41.305	-0.138	-0.175	-3.100**
	guilt	0.397	0.157	0.152	29.286	-0.069	-0.109	-2.148*

Note. SWB= subjective well-being; *Correlation is significant at the .05 level (two tailed); **Correlation is significant at the .01 level (two tailed).

Table S16

Table S16. Re-analysis: the test of mediating effect of use of mobile social-media, boredom proneness and SWB

Mediator	Effect	Effect size	Effect ratio	Boot SE	BootCI LL	BootCI UL
Boredom proneness	Total effect	-0.3840***		0.0417	-0.4659	-0.3021
	Direct effect	-0.1397***		0.0402	-0.2187	-0.0607
	Indirect effect	-0.2443***	63.63%	0.0344	-0.3147	-0.1797

Note. BP= boredom proneness; ***Correlation is significant at the .001 level (two tailed).
